# Transcriptional Variability Associated With CRISPR-Mediated Gene Replacements at the *Phytophthora sojae Avr1b-1* Locus

**DOI:** 10.3389/fmicb.2021.645331

**Published:** 2021-03-18

**Authors:** Biao Gu, Guangda Shao, Wenxin Gao, Jianqiang Miao, Qinhu Wang, Xili Liu, Brett M. Tyler

**Affiliations:** ^1^State Key Laboratory of Crop Stress Biology for Arid Areas and College of Plant Protection, Northwest A&F University, Yangling, China; ^2^Department of Botany and Plant Pathology and Center for Genome Research and Biocomputing, Oregon State University, Corvallis, OR, United States

**Keywords:** *Phytophthora sojae*, RXLR effector, clustered regularly interspaced short palindromic repeat, gene replacement, transcriptional variation

## Abstract

Transcriptional plasticity enables oomycetes to rapidly adapt to environmental challenges including emerging host resistance. For example, the soybean pathogen *Phytophthora sojae* can overcome resistance conferred by the host resistance gene *Rps1b* through natural silencing of its corresponding effector gene, *Avr1b-1*. With the *Phytophthora* CRISPR/Cas9 genome editing system, it is possible to generate site-specific knock-out (KO) and knock-in (KI) mutants and to investigate the biological functions of target genes. In this study, the *Avr1b-1* gene was deleted from the *P. sojae* genome using a homology-directed recombination strategy that replaced *Avr1b-1* with a gene encoding the fluorescent protein mCherry. As expected, all selected KO transformants gained virulence on *Rps1b* plants, while infection of plants lacking *Rps1b* was not compromised. When a sgRNA-resistant version of *Avr1b-1* was reintroduced into the *Avr1b*-1 locus of an Avr1b KO transformant, KI transformants with a well-transcribed *Avr1b-1* gene were unable to infect *Rps1b*-containing soybeans. However, loss of expression of the incoming *Avr1b-1* gene was frequently observed in KI transformants, which resulted in these transformants readily infecting *Rps1b* soybeans. A similar variability in the expression levels of the incoming gene was observed with AVI- or mCherry-tagged Avr1b-1 constructs. Our results suggest that *Avr1b-1* may be unusually susceptible to transcriptional variation.

## Introduction

*Phytophthora sojae* causes destructive root and stem rot diseases of soybeans and has been a model for molecular genetics research into oomycete plant pathogens (Tyler, 2007; Jiang and Tyler, 2012; Wang and Wang, 2018). The pathogenic mechanisms of *Phytophthora* pathogens have been extensively explored since the release of the *P. sojae* genome sequence (Tyler et al., 2006). In particular, hundreds of rapidly evolving effector proteins have been identified as playing a central role in manipulating host defenses and aiding pathogen infection and proliferation (Tyler and Gijzen, 2014; Wang Y. et al., 2019). Among those diverse infection-associated factors, a large superfamily of small secreted hydrophilic proteins with a conserved RXLR motif (RXLR effectors) has been characterized as key contributors to virulence by *P. sojae* and other oomycete plant pathogens (Dou et al., 2008a, b; Jiang et al., 2008; Jiang and Tyler, 2012; Wang and Wang, 2018; Wang Y. et al., 2019). The same superfamily includes most oomycete avirulence (Avr) determinants, which are proteins that enable recognition by host intracellular receptors encoded by major disease resistance (*R*) genes (Tyler and Gijzen, 2014). Recognition of avirulence determinants results in a vigorous defense response that often includes programmed cell death, called a hypersensitive response (HR) (Jones and Dangl, 2006). Most RXLR effector genes display transcriptional dynamics during host infection and other developmental stages (Wang et al., 2011; Ah-Fong et al., 2017).

The *P. sojae* RXLR effector Avr1b, encoded by the first cloned *Avr* gene of oomycete pathogens, *Avr1b*-1 (Shan et al., 2004), has been used as a probe in *Phytophthora* functional genomics (Dou et al., 2008a, b). Avr1b can be recognized by plants carrying the soybean resistance genes *Rps1b* or *Rps1k* in a gene-for-gene manner, triggering HR and leading to failure of infection (Song et al., 2013). Avr1b is capable of suppressing plant programmed cell death triggered by the mouse BAX protein (Dou et al., 2008b) and by other effector proteins (Wang et al., 2011). Constitutive expression of *Avr1b-1* enhances the virulence of *P. sojae* (Dou et al., 2008b). Avr1b-mCherry fusion proteins mainly accumulated around haustoria during infection (Liu et al., 2014) and Avr1b can enter inside plant cells (Dou et al., 2008a; Kale et al., 2010; Tyler et al., 2013). Due to the existence of *P. sojae* natural isolates virulent on *Rps1b* plants, the *Avr1b-1* gene was thought not to be essential for full virulence of *P. sojae* (Shan et al., 2004; Cui et al., 2012). One hypothesis is that paralogous RXLR genes may be functionally redundant with *Avr1b*-1. Another possibility is that numerous RXLR effectors quantitatively contribute to pathogenicity; thus, the variations in virulence from loss of one gene cannot be easily detected.

RXLR effector genes generally reside in gene-sparse regions of oomycete genomes (Tyler et al., 2006; Haas et al., 2009; Baxter et al., 2010). Diverse polymorphisms of those genes have been reported at both the DNA and RNA levels (Wang et al., 2011; Wang Q. et al., 2019). Besides various DNA sequence mutations, the loss of expression of RXLR effector genes encoding avirulence determinants has been described as a mechanism of *P. sojae* for overcoming a number of soybean resistance genes (Shan et al., 2004; Tyler and Gijzen, 2014; Chen et al., 2018; Wang Q. et al., 2019). For example, several virulence-conferring alleles of the *P. sojae Avr1a*, *Avr1b*, *Avr1c*, and *Avr3a/5* genes displayed reduced transcript levels but had unchanged sequences relative to the respective avirulence-conferring alleles (Shan et al., 2004; Qutob et al., 2009, 2013; Dong et al., 2011). In the case of *P. sojae Avr3a*, the gene has been shown to undergo transgenerational gene silencing, resulting in loss of the avirulence-conferring phenotype (Qutob et al., 2013). Both histone methylation-induced transcriptional gene silencing (Wang et al., 2020) and double-stranded RNA-mediated posttranscriptional silencing (Wang Q. et al., 2019) have been proposed to be responsible for natural silencing of *Avr1b-1* in the isolate P6497.

Recently, a *Phytophthora*-specific clustered regularly interspaced short palindromic repeat (CRISPR)/Cas9-mediated genome editing tool was developed for efficient gene knock-out (KO) and replacement (knock-in; KI) in *P. sojae via* either non-homologous end-joining (NHEJ) or homology-directed recombination (HDR) (Fang and Tyler, 2016). Soon after, an “all-in-one” system was developed by inserting the Cas9 and single guide RNA (sgRNA) cassette into one construct with a single neomycin phosphotransferase II (npt II) selection marker that substantially enhanced the efficiency of heritable genome modifications (Fang et al., 2017). As a result, knock-out and complementation of target genes have become basic requirements for functional genomics research in *P. sojae*. The tool has subsequently been deployed in other *Phytophthora* species including *P. capsici* (Chen et al., 2019; Wang W. et al., 2019), *P. palmivora* (Gumtow et al., 2018), *P. parasitica* (Zhang et al., 2020), and *P. litchii* (Situ et al., 2020), as well as in the animal pathogenic oomycete *Aphanomyces invadans* (Majeed et al., 2018).

In this study, we employed the *Phytophthora*-specific CRISPR/Cas9 system to knock out the RXLR effector gene *Avr1b-1* of *P. sojae* and to evaluate the pathotype of *Avr1b-1*-deletion transformants using qualitative and quantitative inoculation assays. The results confirmed that *Avr1b-1* is essential for avirulence of *P. sojae* on *Rps1b* soybean plants but not essential for full virulence. Reintroduction of sgRNA-resistant versions of *Avr1b*-1 was also completed using the same genome editing tool, in combination with a novel strategy for repeated reuse of the G418 resistance selection marker. All KI transformants carrying well-transcribed *Avr1b*-1 genes exhibited restored avirulence phenotypes on *Rps1b* plants. More interestingly, CRISPR-mediated gene replacements into both *Avr1b-1* deletion transformants and wild-type isolate produced large variations in transcript levels of *Avr1b-1*, suggesting that the *Avr1b*-1 locus may be unusually susceptible to transcriptional polymorphisms.

## Materials and Methods

### *P. sojae* Isolates and Culture Conditions

*P. sojae* strains P6497 and P7063 were routinely cultured and maintained on clarified V8 agar plates (10% V8 juice with 0.01% w/v CaCO_3_ and 0.002% w/v β-sitosterol) at 25°C in the dark. Zoospores were induced and harvested from approximately 1 week-old cultures grown on clarified V8 agar or in clarified V8 broth, by flooding the plates or mycelia with sterile distilled water, followed by filtering through 50-mm nylon mesh to remove mycelial fragments (Fang and Tyler, 2016). *P. sojae* transformants were incubated in 12-well plates (Corning, NY, United States) containing V8 medium supplemented with 50 μg/ml G418 for 3–5 days before small-scale genomic DNA extraction. All transformants were routinely grown on clarified V8 plates with or without appropriate antibiotics for successive transfer.

### Soybean Culture Conditions and Inoculation

Soybean plants were grown as described previously (Dou et al., 2008b) with some modifications. Seedlings were grown in a growth chamber with 16 h light at 28°C and 8 h darkness at 25°C. For inoculation assays, 1 week-old seedlings (used for hypocotyl inoculation) or unifoliate leaves of 9–12 day-old seedlings were inoculated with 10–20 μl *P. sojae* zoospore suspension (∼300 zoospores) and maintained in the growth chamber with 100% humidity. For hypocotyl inoculation assays, differences between numbers of surviving plants from Williams (*rps1b*) and L77-1863 (*Rps1b*) cultivars were compared using Fisher’s exact test with a *P*-value cutoff of 0.01. Strains showing significant differences between Williams (*rps1b*) and L77-1863 (*Rps1b*) cultivars were judged as avirulent. Quantitative virulence of *P. sojae* transformants was determined by measuring the lesion size. We analyzed 10 leaves within each treatment, and each treatment was repeated at least three times. Statistical analyses were performed by the Wilcoxon rank sum test with a cutoff of *P* < 0.001.

### sgRNA Design and Plasmid Construction

sgRNAs were designed as described by Fang et al. (2017). In total, six synthesized DNA fragments encoding *Avr1b*-targeted and *mCherry*-targeted sgRNAs were individually inserted into the *Bsa*I and *Nhe*I sites of “all-in-one” CRISPR/Cas9 vector pYF515 using annealing and ligation steps. The cassette harboring each sgRNA was verified by colony-PCR and Sanger sequencing. All HDR constructs contain an upstream flanking region, Avr1b or mutant’s sequence, and a downstream flanking region. Three fragments of interest were inserted into the *Eco*RI and *Hin*dIII restriction sites of pBluescript II KS *via* In-Fusion Cloning (TaKaRa, China). PCR amplification of the 1-kb upstream and downstream flanking ends was conducted with primer pairs Fl-Avr1b/Rl-Avr1b and Fr-Avr1b/Rr-Avr1b. PCR products of Avr1b or mCherry were obtained with primer pairs PsF/PsR and 1bmcF/1bmcR. The sgRNA-resistant version of Avr1b (Avr1b^sgR^) was synthesized from the company (GeneCreate, China) with multiple synonymous substitutions in three sgRNA targeting sites (Supplementary Figure S4A). The biotin-acceptor AVI-tag was fused to the C-terminus of Avr1b^sgR^ by PCR using primer pairs PsF/Ps4WTR1/Ps4WTR2. The Avr1b^sgR^-mCherry fusion was obtained *via* fusion PCR using primer pairs PsF/1bmcR, 1bmcR/R-mcR, and PsF/R-mcR. The Avr1b^sgR^-AVI or Avr1b^sgR^-mCherry was used to replace Avr1b or mCherry in *P. sojae* transformation (Supplementary Figure S1). The vector for particle bombardment-mediated transient expression was as described by Dou et al. (2008b). Avr1b or Avr1b^sgR^ was amplified with primer 1b-F/1b-R and was inserted into the *Sma*I and *Kpn*I restriction sites of pUC1b *via* T4 DNA ligation (TaKaRa, China). High concentrations of plasmids for bombardment were prepared as described by Zhao et al. (2020). Standard molecular techniques were performed according to the instructions from kit manufacturers. The oligonucleotides used in this study are listed in Supplementary Table S3.

### Transformation of *P. sojae*

Polyethylene glycol-mediated transformations were performed as reported previously (Fang et al., 2017). Specifically, young mycelia were collected by inoculating 30 ml of nutrient pea broth in 90 × 20 mm petri dishes with 30–40 thin agar disks (each 0.5 cm in diam.). Mycelial mats were rinsed twice with sterilized H_2_O and 0.8 M mannitol, followed by a 5 min wash in 0.8 M mannitol before enzyme digestion. Protoplasts were generated with a combination of 1% Trichoderma Lysing Enzymes (Sigma, China) and 0.1% CELLULYSIN^®^ Cellulase (Merck, China) in Fry buffer (0.4 M mannitol, 20 mM KCl, 20 mM MES, pH 5.7, 10 mM CaCl_2_). The density of protoplasts was monitored to ensure that a minimum of 1 × 10^7^ per ml was achieved. About 30–40 μg of plasmid DNA was used for transformation. Protoplasts were regenerated overnight in pea broth containing 0.4 M mannitol. Regenerated protoplasts were collected and distributed onto plates of regeneration media containing 50 μg/ml of G418 for 2–3 days. Half of the G418-insensitive colonies were transferred to 12-well plates containing V8 broth supplemented with 50 μg/ml of G418 and incubated for 3–4 days at 25°C before DNA extraction. The other half were routinely subcultured on V8 agar plates without antibiotics.

### Zoospore Isolation

To obtain homokaryotic colonies, zoospore purification was routinely performed for selected transformants as described by Fang et al. (2017) with some modifications. Briefly, fresh cultures of PCR-verified transformants were flooded with sterile dH_2_O 6–8 times. Then, the mycelial colony (60 mm plate) was covered with 3 ml sterile dH_2_O, and zoospore release usually began after 3–4 h of room temperature incubation. Zoospore suspensions were diluted to 1 × 10^3^ per milliliter. Zoospore drops (1 μl per drop) were loaded onto V8 plates and incubated at 25°C for no more than 24 h. With a light microscope, single zoospore-derived mycelial colonies were selected and transferred to a new V8 plate for further culturing.

### DNA and RNA Isolation and cDNA Synthesis

Total gDNA was extracted as described by Fang and Tyler (2016). Total RNAs were extracted from zoospore-inoculated hypocotyls or unifoliate leaves with the RNeasy Plant Mini Kit (Qiagen, Germany). Both oligo-(dT)_18_ and random primer mix were used for first-strand cDNA synthesized by SuperScript^TM^ III (Thermo Fisher, China) according to the product instructions.

### Semiquantitative and Quantitative PCR Analysis

Semiquantitative RT-PCR was carried out with 1 μl of fivefold diluted cDNA products in a 20 μl volume containing Taq PCR Mastermix (Tiangen, Beijing, China) and matching primers. PCR conditions consisted of one cycle of 95°C for 3 min, followed by 30 cycles of a three-step procedure (1 min at 95°C, 1 min at 56°C, and 1 min at 72°C) and a final step of 5 min at 72°C. Quantitative PCR of genomic DNA for biomass measurements and quantitative reverse transcription PCR (qRT-PCR) for transcript measurements were performed as previously described (Gu et al., 2020) using CFX connect (Bio-Rad, CA, United States). Genomic DNA levels or gene expression levels relative to the reference gene were calculated with the 2^–ΔΔCT^ method. The *Actin* genes of *P. sojae* and soybean were chosen as the reference genes (Wang et al., 2011).

### Detection and Validation of Targeted Mutagenesis

PCR amplifications using Taq DNA polymerase (TaKaRa, Japan) were conducted to detect the targeted mutations in transformants. Approximately 10 ng of gDNA was used as PCR template. To detect HDR events in individual transformants, primers located outside the *Avr1b* homology arms and within the *Avr1b-1* locus were used. For screening homozygous transformants, PCR was performed with primers spanning from outside the homology arms to within the *Avr1b-1* or *mCherry* coding regions.

### Transient Expression by Particle Bombardment of Soybean Leaves

The unifoliate leaves of soybean seedlings were selected for bombardment using a double-barreled extension of the Bio-Rad He/1,000 particle delivery system (Kale and Tyler, 2011). Plasmid DNA was isolated with the EndoFree Maxi Plasmid Kit (Tiangen, China) and concentrated to 5–6 mg/ml in sterile H_2_O. Tungsten particles were prepared as reported previously (Dou et al., 2008a). Plasmids encoding beta-glucuronidase (GUS) mixed with a *GFP* control or a mixture of plasmids encoding Avr1b proteins and GUS were delivered into host cells side by side *via* the double barrel gene gun. Parameters were set as reported (Gu et al., 2011). After bombardment, the leaves were incubated for 2–3 days in darkness at 25°C and then stained for at least 4 h at 37°C in GUS staining solution. Blue spots were counted under a dissecting microscope at × 8– × 160 magnification. For each paired shot (*GUS* + *Avr1b* or *Avr1b*^*sgR*^ vs. *GUS* + *GFP*), the logarithm of the ratio of the spot numbers with the fusion protein compared with that of the control was calculated, and then the log ratios obtained from the *Rps*1b and *rps*1b leaves were compared by the Wilcoxon rank sum test (Kale and Tyler, 2011).

### Confocal Microscopy

A laser scanning confocal microscope FV3000 (Olympus, Japan) was used to examine the expression and subcellular localization of mCherry or mCherry-fused proteins in transformants upon infection. Living hyphae or zoospores were inoculated onto the hypocotyls of etiolated soybean seedlings grown for 7–10 days in darkness. Images were captured using a × 63 oil objective with excitation/emission settings of 561 nm/570–630 nm for mCherry. Calibrations of gain settings were performed with control hypocotyls as a range of background fluorescence to avoid capturing background fluorescence.

## Results

### *Avr1b-1* KO Transformants Fail to Trigger HR on *Rps1b* Soybean Plants

**FIGURE 1:**
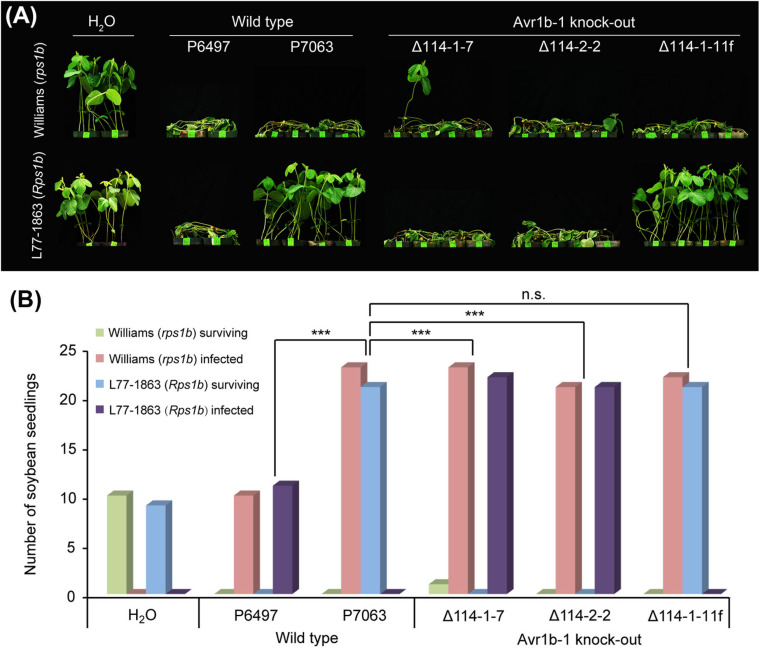
Avirulence phenotypes of *Avr1b-1* knock-out transformants measured by hypocotyl inoculations. **(A)** *Avr1b-1*-silenced wild-type isolate P6497, *Avr1b-1-*expressing wild-type isolate P7063, and two homozygous *Avr1b-1* deletion transformants Δ114-1-7 and Δ114-2-2, plus a non-replacement transformant Δ114-1-11f were inoculated onto the hypocotyls of 7 day-old seedlings of soybean cultivar Williams (*rps1b*) or L77-1863 (*Rps1b*). Pictures were taken 3 days after wound inoculation. **(B)** Numbers of infected and surviving seedlings after inoculation with the *P. sojae* strains. A strain was deemed to be avirulent if the number of inoculated *Rps1b* seedlings surviving was significantly higher than the number of surviving seedlings without the *Rps1b* gene, and the number was not significantly different from the number of surviving seedlings inoculated with wild-type P7063. At least eight seedlings were used for the inoculation of each control or transformant. The significance of differences was determined by Fisher’s exact test (*P* < 0.001). *** = significant; n.s. = not significant.

To confirm that *Avr1b-1* is essential for conferring HR on soybean plants carrying *Rps1b*, we employed CRISPR/Cas9-dependent mutagenesis to delete *Avr1b-1* by gene replacement. The *P. sojae* isolate P7063 carries a single copy of *Avr1b-1* and lacks the close paralog *Avh1*; this strain was used as the recipient strain for *Avr1b-1* replacement. Based on *in silico* analysis, three sgRNAs matching different locations in the *Avr1b-1* coding region were designed to guide Cas9 cleavage that would trigger homology direct repair (HDR) (Supplementary Figure S1 and Supplementary Table S1). One of the three sgRNAs, sg114R, produced the highest editing efficiency, with an average frequency of homozygous replacement events of 45%. In comparison, sg19 and sg285 yielded only a few homozygous transformants. Following PCR verification (Supplementary Figure S2A), two independent homozygous transformants Δ114-1-7 and Δ114-2-2, together with one non-replacement transformant Δ114-1-11f, were selected for further analysis. No abnormalities in morphology or fertility were observed in the selected mutants (Supplementary Table S2). All genes in the *Avr1b-1* locus (either *Avr1b-1* itself or *mCherry*) were normally transcribed during infection according to semiquantitative RT-PCR analysis (Supplementary Figure S2B). Next, the transformants, along with wild-type isolates P6497 and P7063, were subjected to avirulence analysis on soybean plants with or without the *Rps1b* gene. As shown in Figure 1, the Avr1b-1 KO transformants Δ114-1-7 and Δ114-2-2, plus P6497, which is naturally silenced at *Avr1b-1*, successfully infected the *Rps1b* soybean seedlings, suggesting that *Rps1b-*dependent disease resistance was not initiated. In contrast, the non-replacement transformant Δ114-1-11f and the control isolate P7063 failed to infect the *Rps1b* soybean plants. All wild-type isolates and transformants killed *rps1b* soybean seedlings (Figures 1A,B).

**FIGURE 2:**
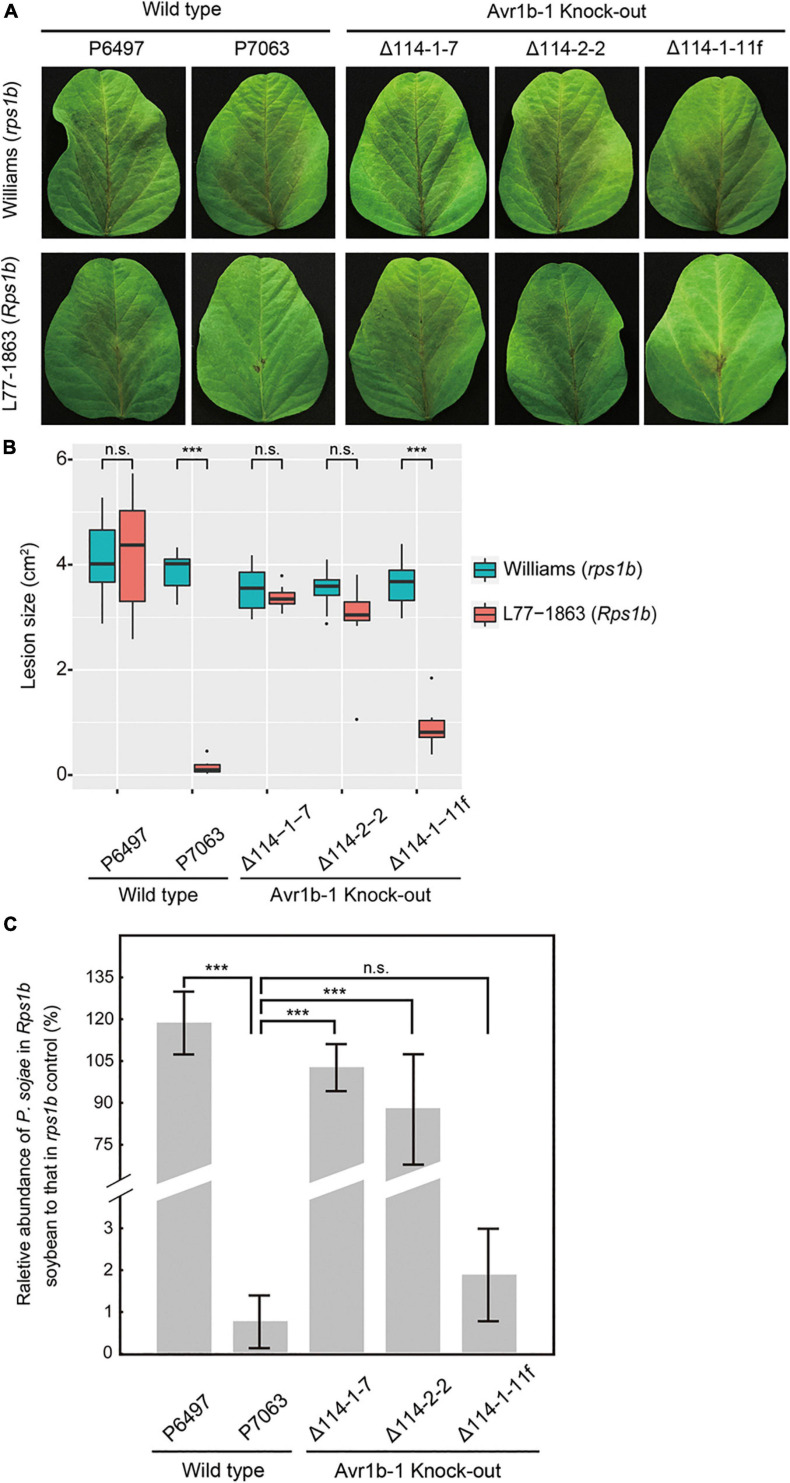
Avirulence phenotypes of *Avr1b-1* knock-out transformants measured by detached leaf inoculations. **(A)** Soybean leaf lesions 3 days after zoospore inoculation of Williams (*rps*) or L77-1863 (*Rps1b*) unifoliate leaves. **(B)** Sizes of lesions after 3 days. Lesions on eight unifoliate leaves were measured in each of three independent experiments. The relative lesion size was measured from photographs with ImageJ software. Lesion sizes were compared between Williams (*rps*) and L77-1863 (*Rps1b*) using the Wilcoxon rank sum test. ***P < 0.001, n.s. *P* > 0.1. **(C)** Avirulence phenotypes of *Avr1b-1* knock-out transformants measured by real-time PCR-based quantification of *P. sojae* genomic DNA in infected tissue. Proliferation of *P. sojae* was plotted as the ratio between the abundance of *PsActin* relative to *GmActin* DNA in infected L77-1863 leaves compared with that in infected Williams leaves, at 72 hpi. Error bars represent standard deviation of three technical replicates. This experiment was replicated three times. Error bars represent the mean ± standard deviation. *P*-values were determined by the Wilcoxon rank sum test; *** indicates *P* < 0.001.

To quantitatively measure the resistance in L77-1863 (*Rps1b*) or Williams (*rps1b*) soybean cultivars, we inoculated soybean leaves with zoospores and monitored the lesion progression. The rate of lesion progression on the unifoliate leaves was measured, and no statistically significant differences were observed for any of the isolates and transformants infecting Williams (*rps1b*) soybean leaves (Figure 2). However, on L77-1863 leaves (containing *Rps1b*), strains P6497, Δ114-1-7, and Δ114-2-2 formed lesions that were significantly larger than the lesions produced by control strains P7063 and Δ114-1-11f (Figures 2A,B). Moreover, when the relative biomass of the pathogen was measured at 72 h post-inoculation (hpi) as genomic DNA ratios by qPCR, the biomasses of the *Avr1b-1* KO mutants were about 45—110-fold greater on *Rps1b* soybean L77-1863 than those of the *Avr1b-1*-expressing transformant and P7063 control (Figure 2C). These results were consistent with the hypocotyl infection assay and confirmed that *Avr1b-1* is necessary and sufficient for *Rps*1b recognition to trigger HR.

### *Avr1b-1* Is Not Required for Full Virulence of *P. sojae*

**FIGURE 3:**
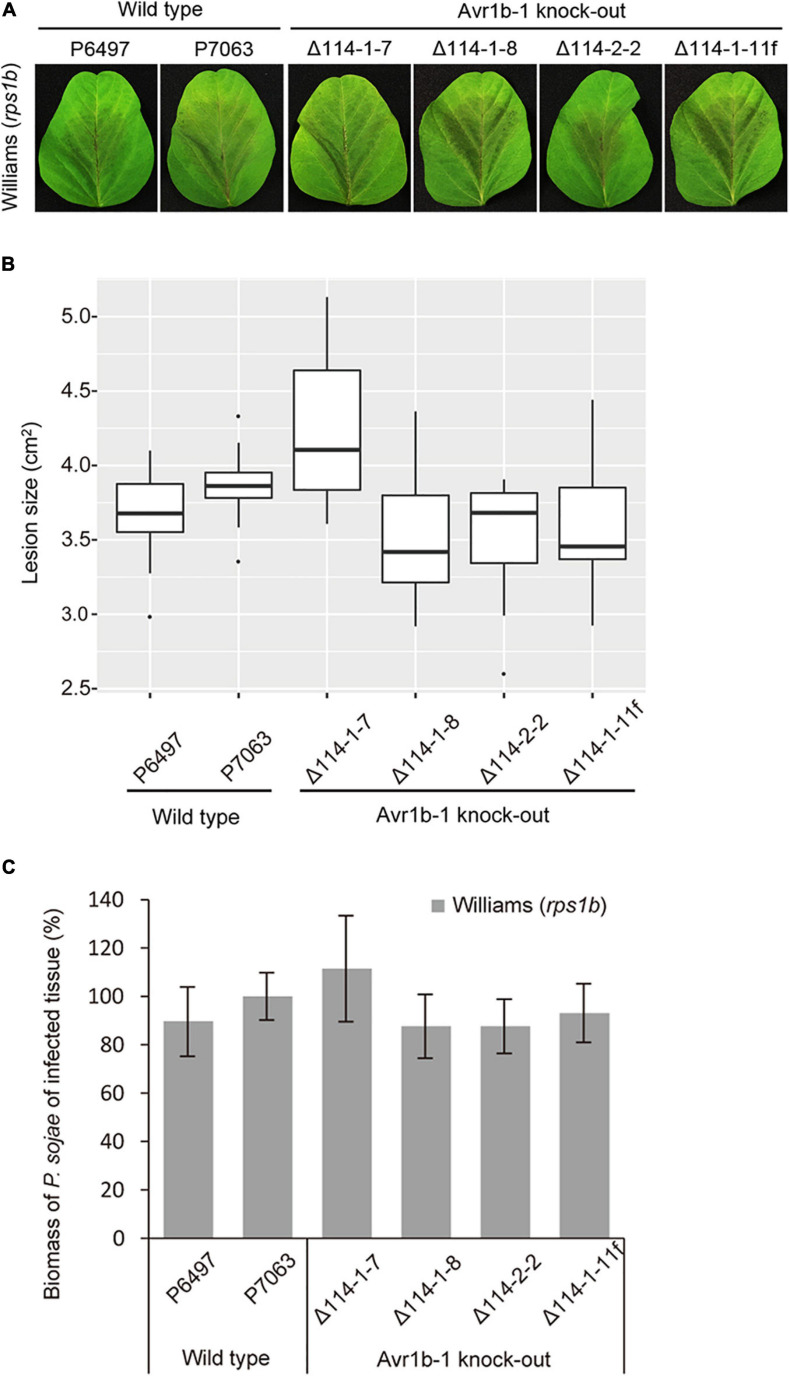
Virulence phenotypes of *Avr1b-1* knock-out transformants on Williams soybean leaves. **(A)** *Avr1b-1*-silenced isolate P6497; *Avr1b-1*-expressing isolate P7063; three homozygous *Avr1b-1* deletion transformants Δ114-1-7, Δ114-1-8, and Δ114-2-2; plus non-HDR transformant Δ114-1-11f were inoculated onto unifoliate leaves of Williams (*rps1b*). Pictures were taken 3 days after inoculation. **(B)** Average lesion sizes determined from at least 10 inoculated unifoliate leaves in each of three independent experiments. The relative lesion sizes were measured by ImageJ software. Error bars represent the mean ± standard deviation. *P*-values were determined by the Wilcoxon rank sum test. **(C)** Virulence phenotypes of *Avr1b-1* knock-out transformants measured by real-time PCR-based quantification of *P. sojae* genomic DNA in infected tissue. Error bars represent standard deviation of three technical replicates. This experiment was replicated three times. Error bars represent the mean ± standard deviation.

Overexpression of *Avr1b-1* in *P. sojae* confers increased virulence on soybean plants compared with the recipient strain (Dou et al., 2008b), but natural loss of *Avr1b-1* transcripts does not compromise the virulence of *P. sojae* strain P6497 (Shan et al., 2004). To definitively confirm this result in a strain that normally expresses *Avr1b-1*, we measured the virulence of the P7063 KO mutants. P7063 and P6497, together with KO transformants Δ114-1-7, Δ114-1-8, Δ114-2-2, and non-replacement transformant Δ114-1-11f were assessed by zoospore inoculation of the leaves. Based on the lesion sizes, no significant virulence differences were observed among the six strains (Figures 3A,B). Furthermore, relative biomasses of the *Avr1b-1* KO mutants measured on soybean plants at 72 hpi were similar to those produced by wild-type strains P7063 and P6497 and the non-replacement transformant (Figure 3C). These results demonstrated that the RXLR effector gene *Avr1b-1* was not required for the full virulence of strain P7063 under the conditions of this assay.

### Repeated Use of Neomycin Phosphotransferase II (npt II) as a Selection Marker for Complementation

**FIGURE 4:**
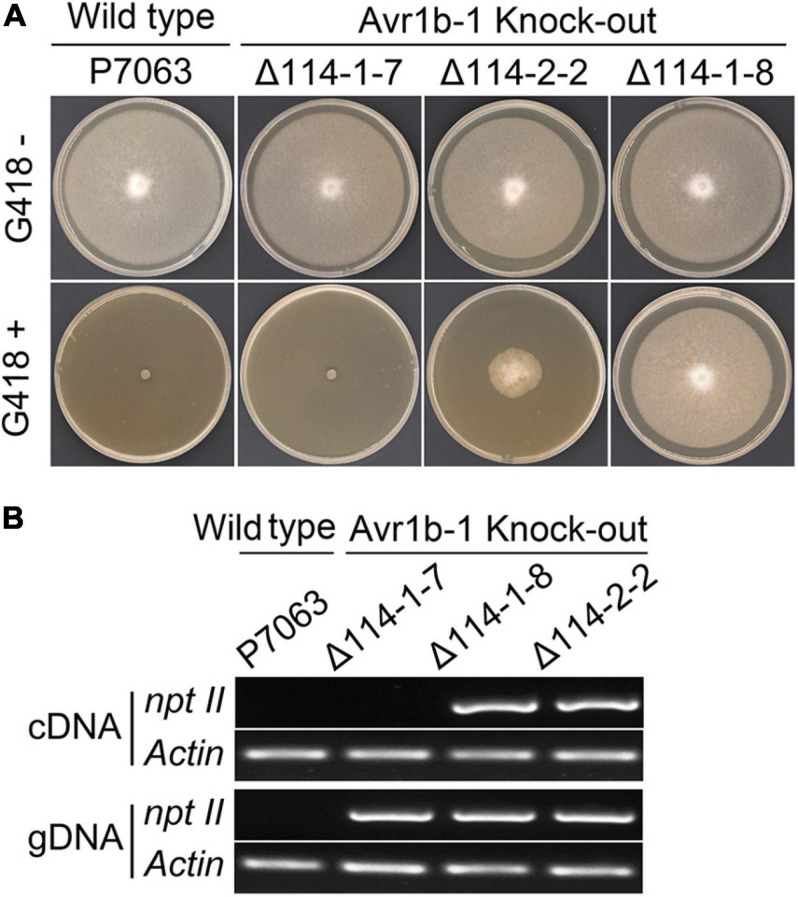
G418 sensitivity of *Avr1b-1* knock-out transformants. **(A)** Wild-type P7063 and three selected *Avr1b-1* deletion transformants cultured on clarified V8 medium with or without 50 μg/ml G418. Transformants Δ114-1-7 and Δ114-2-2 were isolated from G418 regeneration medium and then subcultured in V8 medium. Transformant Δ114-1-8 was maintained on V8 medium containing 50 μg/ml G418. **(B)** PCR detection of *npt II* sequences in genomic DNA and RNA of *Avr1b-1* knock-out transformants and P7063 with primer pair qNptF/R. *P. sojae Actin* was used as the reference gene.

We noticed that successive subculture of the homozygous *Avr1b-1* KO transformants in the absence of the antibiotic G418, used for the initial transformation, resulted in loss of G418 resistance. Most of the transformants, including Δ114-1-7, could not survive in media containing 50 μg/ml G418, while a smaller fraction of transformants, such as Δ114-2-2, retained weak growth in G418 media (Figure 4A). This observation raised the possibility that G418 could be used as a selection marker in complementation experiments using G418-sensitive transformants as recipients. The *npt II* genes in those G418-sensitive and semisensitive transformants were analyzed at both the DNA and RNA levels. As shown in Figure 4B, the *npt II* gene could be detected in genomic DNA from all transformants, but *npt II* was not detected in the control strain P7063 (with *Actin* as a positive control). When the *npt II* transcript levels were assessed, *npt II* could be detected in a transformant Δ114-1-8, which retained G418 resistance, and in Δ114-2-2, which retained weak G418 resistance. However, transcripts of *npt II* could not be detected in the G418-sensitive transformant Δ114-1-7 nor in the wild-type control, P7063. Thus, it appeared that the *npt II* gene had become partially or fully silenced in Δ114-2-2 and Δ114-1-7, respectively. To test if the *npt II* silencing in these two KO lines was stable enough for G418 selection following transformation and to test if the silencing of the ectopic *npt II* genes could be overcome by incoming plasmid DNA carrying *npt II*, we used both Δ114-1-7 and Δ114-2-2 as recipients to perform protoplast regeneration and transformation with a construct carrying mCherry-tagged Avr1b. Using the partially silenced Δ114-2-2 as the recipient resulted in numerous false-positive colonies on G418-containing plates during regeneration (Supplementary Figures S3A,B). In contrast, when Δ114-1-7 was used as a recipient, no G418-resistant colonies were observed in the absence of *npt II*-containing plasmid DNA. On the other hand, genuine transformants could be obtained using *npt II*-containing plasmids (Supplementary Figure S3B), indicating that silencing of the resident *npt II* gene did not affect the expression of incoming *npt II* genes. These results indicated that full loss of G418 resistance resulting from complete silencing of *npt II* in the recipient transformant was required for successful gene complementation when using G418 as a selection marker for the second time.

### sgRNA-Resistant Version of *Avr1b-1* for Complementation Experiments

**FIGURE 5:**
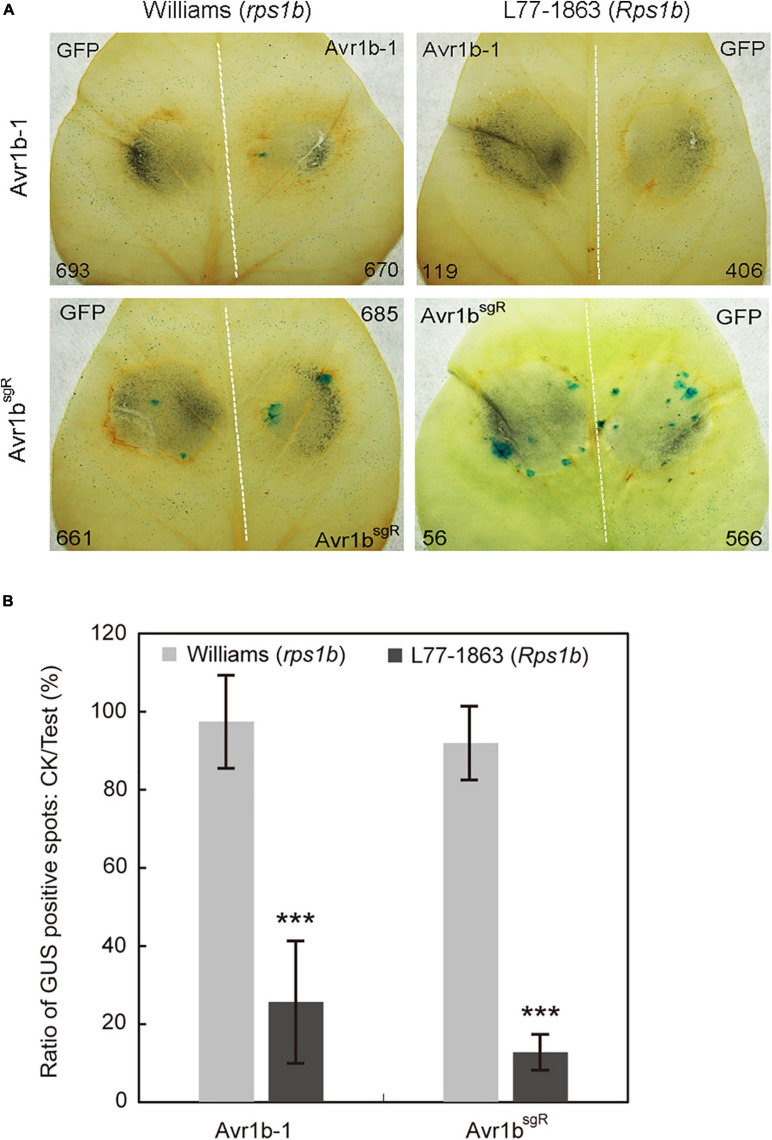
Transient expression of *Avr1b-1^*sgR*^* leads to cell death in *Rps1b* soybean leaves. **(A)** Soybean leaves from Williams (*rps1b*) and L77-1863 (*Rps1b*) were subjected to double-barreled particle bombardment with pairs of samples containing GUS reporter DNA plus either Avr1b^sgR^ DNA or *GFP* control DNA. After staining, the numbers of blue spots on Williams (*rps1b*) and L77-1863 (*Rps1b*) leaves were counted. A functional *Avr1b-1* gene is expected to significantly reduce the number of blue spots on L77-1863 (*Rps1b*) leaves, but not Williams (*rps1b*) leaves, due to triggering *Rps1b*-mediated cell death. The white dotted line separates the two bombardment sites. Numbers of GUS spots counted are indicated. **(B)** Ratios between the numbers of GUS spots produced in the presence of *Avr1b-1* vs. control *GFP* DNA on each leaf, averaged from 10 soybean leaves, in a single experiment. Error bar represents the mean ± standard deviation. The significance of the difference between Williams and L77-1863 leaves was determined by the Wilcoxon rank sum test (Gu et al., 2011), ***P < 0.001.

As a prelude to conducting *Avr1b-1* complementation experiments, we introduced synonymous substitutions at sgRNA binding sites in *Avr1b-1* to prevent the potential cleavage of the incoming DNA by the previously introduced Cas9 protein in the transformants (Supplementary Figure S4A). To validate whether the sgRNA-resistant version of *Avr1b-1* (*Avr1b*^*sgR*^) could be recognized by *Rps1b*, we used double-barreled particle bombardment (Dou et al., 2008a, b; Kale and Tyler, 2011) to co-express either *Avr1b-1* or *Avr1b*^*sgR*^ with a GUS reporter in *rps1b* and *Rps1b* soybean leaves. Compared with the GFP control, the number of surviving GUS-positive (blue) patches was reduced by about 80% in *Rps1b* soybean leaves in the presence of *Avr1b-1* or *Avr1b*^*sgR*^, indicating that HR was activated in the soybean cells (Figure 5). In *rps1b* soybean leaves, *Avr1b-1* and *Avr1b*^*sgR*^ produced no reduction of GUS blue spots compared with GFP, indicating that no HR occurred (Figure 5B). These results confirmed that *Avr1b*^*sgR*^ could produce a functional Avr1b gene product.

### Reintroduction of *Avr1b*^*sgR*^ Into Deletion Transformants Restores Their Avirulence Phenotypes on Soybean Plants Containing *Rps1b*

**FIGURE 6:**
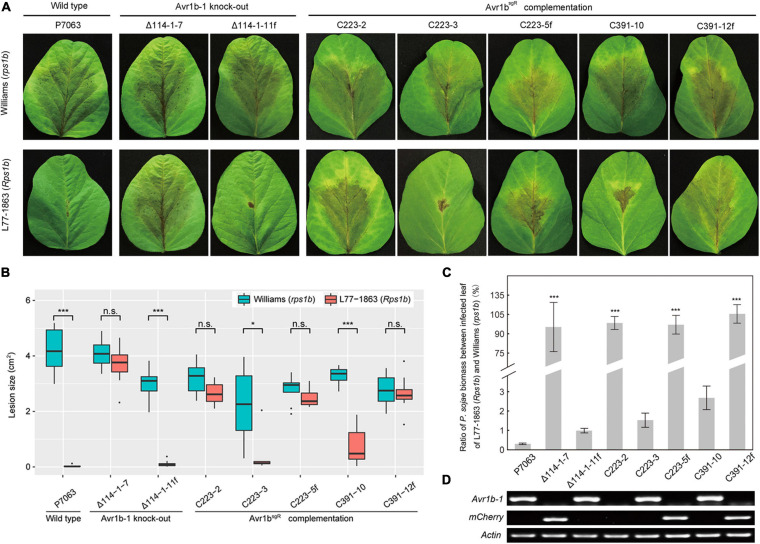
Evaluation of avirulence activity of *Avr1b*^*sgR*^ knock-in transformants. **(A)** *Avr1b*^*sgR*^ knock-in transformants (C223-3 and C391-10) and control strains (wild-type P7063, Δ114-1-7, Δ114-1-11f, C223-5f, and C391-12f) were tested for avirulence by zoospore inoculation onto unifoliate leaves of Williams (*rps1b*) and L77-1863 (*Rps1b*). Pictures were taken 3 days after inoculation. **(B)** Average lesion sizes on the leaves of Williams (*rps1b*) and L77-1863 (*Rps1b*) measured on 10 unifoliate leaves from each of three independent experiments. Sizes were measured with ImageJ software. Error bars represent the mean ± standard deviation. *P*-values were calculated with Wilcoxon rank sum test, * and *** indicate *P*-value < 0.05 and < 0.001, respectively. **(C)** Avirulence phenotypes of *Avr1b-1* knock-in transformants measured by real-time PCR-based quantification of *P. sojae* genomic DNA in infected tissue. Proliferation of *P. sojae* is plotted as the ratio between abundance of *PsActin* relative to *GmActin DNA* in infected L77-1863 leaves compared with Williams leaves at 72 hpi. This experiment was replicated three times. Error bars represent the mean ± standard deviation. *P*-values were determined by the Wilcoxon rank sum test. ***P < 0.001. **(D)** RT-PCR analysis of *Avr1b-1* and *mCherry* transcripts in *Avr1b-1* knock-in transformants. *P. sojae Actin* was used as the reference gene.

In order to confirm that the deletion of *Avr1b-1* was responsible for the loss of soybean resistance conferred by *Rps1b*, *Avr1b*^*sgR*^ was introduced into *Avr1b* KO transformant Δ114-1-7 by replacement of the *mCherry* gene that was residing at the *Avr1b-1* locus. Three sgRNAs, mc223, mc370, and mc391, targeted to different locations in *mCherry*, were designed to guide Cas9 cleavage and HDR (Supplementary Table S1 and Supplementary Figure S4B). Based on PCR assays (Supplementary Figure S5), three homozygous complementation transformants, C223-2, C223-3, and C391-10, and two non-replacement transformants, C223-5f and C391-12f, were identified. No morphological or fertility changes were observed in any of these *P. sojae* strains. The strains, together with *Avr1b-1* non-replacement transformant Δ114-1-11f and wild-type strain P7063, were subjected to avirulence analysis on soybean plants with or without the *Rps1b* gene. Of the *Avr1b*^*sgR*^ complementation transformants, C223-3 and C391-10 failed to infect *Rps1b* leaves but successfully infected Williams (*rps1b*) leaves, demonstrating that the sgRNA-resistant *Avr1b* gene could complement the *Avr1b-1* KO (Figures 6A,B). The genomic biomass measurements were fully consistent with the lesion sizes on susceptible and resistant plants (Figure 6C). As expected, non-replacement transformants, C223-5f and C391-12f, were fully virulent on *Rps1b* as well as *rps* leaves. Surprisingly, however, homozygous complementation transformant C223-2 was virulent on both *Rps1b* and *rps1b* plants. RT-PCR analysis revealed that C223-2 failed to produce any detectable *Avr1b*^*sgR*^ transcript, whereas *Avr1b*^*sgR*^ transcripts were readily detected in C223-3 and C391-10 as well as in the controls P7063 and Δ114-1-11f (Figure 6D).

### Silencing of the *Avr1b-1* Locus in Gene Replacement Transformants

**FIGURE 7:**
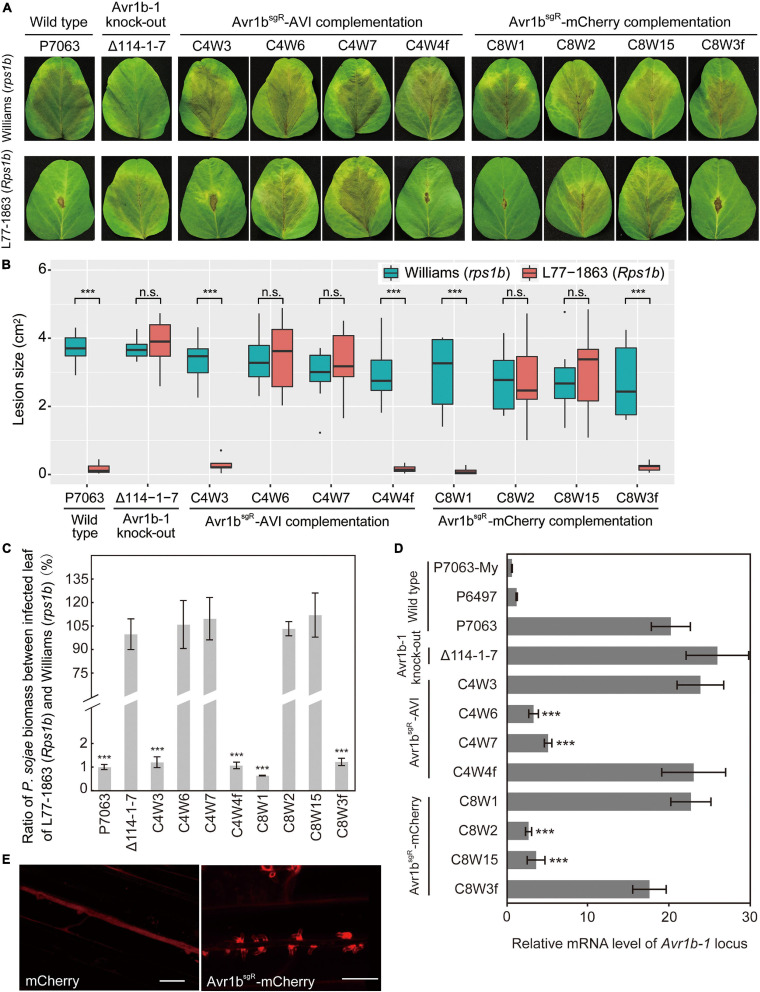
Characterization of *Avr1b-1* knock-in transformants derived from *Avr1b-1^*sgR*^* fusion genes. **(A)** Lesions produced by transformants and control strains. Transformants were *Avr1b^*sgR*^-AVI* KI strains C4W3, C4W6, and C4W7, and *Avr1b^*sgR*^-mCherry* KI strains C8W1, C8W2, and C8W15. Control strains were wild-type P7063, *Avr1b-1* deletion transformant Δ114-1-7, *Avr1b^*sgR*^-AVI* failed KI strain C4W4f, and *Avr1b^*sgR*^-mCherry* failed KI strain C8W3f. Zoospores from each strain were inoculated onto unifoliate leaves of soybean cultivar Williams (*rps1b*) or L77-1863 (*Rps1b*). Pictures were taken 3 days after zoospore inoculation. **(B)** Average lesion sizes on unifoliate soybean leaves inoculated with wild-type or transgenic *P. sojae*, measured from eight leaves in each of three independent experiments. Pictures were taken 3 days after inoculation, then relative lesion sizes were measured with ImageJ software. Error bars represent the mean ± standard deviation. *P*-values were calculated with the Wilcoxon rank sum test, ***P < 0.001. n.s. *P* > 0.1. **(C)** Avirulence phenotypes of *Avr1b-1* knock-in transformants measured by real-time PCR-based quantification of *P. sojae* genomic DNA in infected tissue. Proliferation of *P. sojae* is plotted as the ratio between the relative abundance of *PsActin* DNA to *GmActin* DNA in infected L77-1863 leaves compared with Williams leaves at 72 hpi. This experiment was replicated three times. Error bars represent the mean ± standard deviation. *P*-values were determined with the Wilcoxon rank sum test. ***P < 0.001, n.s. *P* > 0.1. **(D)** Transcript levels produced by genes resident at the *Avr1b-1* locus in wild-type and transgenic lines during soybean infection at 36 hpi. Levels were assayed by qRT-PCR using *Avr1b-1* primers except for Δ114-1-7, for which *mCherry* primers were used (Supplementary Table S3). Transcript levels were normalized against *P. sojae Actin*. Error bars represent the mean ± standard deviation. *P*-values were calculated with the Wilcoxon rank sum test, ***P < 0.001, n.s. *P* > 0.1. RNA samples from the Avr1b-silenced isolate P6497 and *in vitro*-grown P7063 (P7063-My) were analyzed as controls. **(E)** Avr1b-mCherry fusion proteins preferentially accumulate in haustoria. Haustorial accumulation of Avr1b-mCherry during infection of etiolated hypocotyls by C8W1 (right panel) compared with uniform cytoplasmic distribution of mCherry in Δ114-1-7 (left panel). Images were obtained by confocal microscopy at 36 hpi. Scale bars = 20 μm.

In order to produce transformants expressing tagged versions of Avr1b from the *Avr1b-1* locus, for Avr1b function experiments, genes encoding mCherry-tagged or AVI-tagged Avr1b^sgR^ were used to replace *Avr1b-1* in wild-type strain P7063 directly, using *Avr1b-1* sgRNAs, sg114R. In each case, based on PCR screening, 20–45% of the candidates screened were homozygous replacement transformants (Supplementary Figure S6). Three homozygous replacement transformants and one non-replacement transformant were selected for each of Avr1b^sgR^-mCherry (C8W1, C8W2, C8W15, and C8W3f, respectively) and Avr1b^sgR^-AVI (C4W3, C4W6, C4W7, and C4W4f, respectively) and then subjected to virulence tests. Equal concentrations of zoospores produced by individual transformants or wild-type P7063 were inoculated onto both Williams (*rps1b*) and L77-1863 (*Rps1b*) soybean plants. Homozygous transformants C4W3 and C8W1 and the positive controls C4W4f, C8W3f, and P7063 exhibited the expected avirulence phenotype, failing to infect *Rps1b* plants and manifesting an HR (Figures 7A,B). Surprisingly, however, C4W6, C4W7, C8W2, and C8W15 infected *Rps1b* and *rps* leaves equally well, like the negative control, KO strain Δ114-1-7. The lesion size data were confirmed by the genomic biomass measurements on *rps* and *Rps1b* leaves (Figure 7C). Thus, these four strains had lost their avirulence phenotype.

To explore the reasons for loss of avirulence among the transformants, the relative transcript levels of the tagged *Avr1b-1* genes during soybean infection were analyzed by qRT-PCR. As shown in Figure 7D, the transcript levels of *Avr1b-1*, *Avr1b^*sgR*^-AVI*, and *Avr1b^*sgR*^-mCherry* in C4W3 and C8W1 were comparable to those in the positive controls, C4W4f, C8W3f, and P7063, and the *mCherry* gene in Δ114-1-7. In contrast, the *Avr1b-1* transcript levels in transformants C4W6, C4W7, C8W2, and C8W15 were 3–6-fold lower than in the wild type, P7063.

To further examine the expression of *Avr1b^*sgR*^-mCherry* in C8W1 during infection, zoospores from C8W1 were inoculated on the hypocotyls of Williams (*rps1b*) seedlings, then accumulation of the Avr1b-mCherry fusion protein was observed by confocal microscopy. The fusion protein could readily be observed to accumulate specifically around haustoria-like structures, as previously reported for ectopically expressed Avr1b-RFP (Liu et al., 2014). In contrast, when we observed the distribution of mCherry during infection by Δ114-1-7 which expresses free non-secreted mCherry from the *Avr1b-1* locus, the fluorescent protein was uniformly distributed within the mycelium of the transformant (Figure 7E).

To extend the results described above, we tested additional *Avr1b^*sgR*^-AVI* and *Avr1b^*sgR*^-mCherry* gene replacement transformants from new independent transformations. Out of 13 homozygous Avr1b^sgR^-AVI KI transformants and 15 homozygous Avr1b^sgR^-mCherry KI transformants, only two and one transformants, respectively, induced HR on *Rps1b* soybean plants (Table 1), suggesting a very high frequency of silencing of the incoming transgene. The avirulence activities of the homozygous transformants were confirmed to be associated with normal transcript levels of either Avr1b^sgR^-AVI or Avr1b^sgR^-mCherry, respectively, whereas *Avr1b*^*sgR*^ transcripts were not detected from the virulent transformants (Supplementary Figure S7). When the *Avr1b-1* deletion mutant Δ114-1-7, which expresses *mCherry* at the *Avr1b-1* locus, was used as a recipient for gene replacements with *Avr1b^*sgR*^-AVI* or *Avr1b^*sgR*^-mCherry*, failed expression was also observed at high frequency, based both on assays of avirulence (Table 1) and transcript presence (Supplementary Figure S7). Of the *Avr1b^*sgR*^-mCherry* homozygous replacement transformants, 9/11 showed failed expression based on inoculation of *Rps1b* soybean leaves and RT-PCR tests, while 9/9 *Avr1b^*sgR*^-AVI* homozygous replacement transformants exhibited failed expression (Table 1 and Supplementary Figure S7). These results suggest that the *P. sojae Avr1b-1* locus is highly susceptible to loss of expression as a result of CRISPR/Cas9-mediated gene replacements.

**TABLE 1:** Silencing of *Avr1b-1* locus in homozygous *Avr1b*^*sgR*^ knock-in transformants.

Recipient^a^	HDR constructs^b^	G418^R^ colonies^c^	Homozygous KI transformants^d^	Avirulent on *Rps1b*	Virulent on *Rps1b*	Silencing of *Avr1b-1* locus^e^
P7063	Avr1b^sgR^-AVI	32	13	2	11	11/13
	Avr1b^sgR^-mCherry	34	15	1	13	13/15
Δ114-1-7	Avr1b^sgR^-AVI	33	11	2	9	9/11
	Avr1b^sgR^-mCherry	30	9	0	9	9/9

*^a^P. sojae wild-type P7063, Avr1b-1 knock-out transformant Δ114-1-7.*

*^b^Homology-directed recombination constructs used to produce Avr1b^sgR^ knock-in transformants, consisting of AVI- or mCherry-tagged Avr1b^*sgR*^ fused to 1 kb flanking sequence at each end.*

*^c^Regenerated colonies exhibiting vigorous growth in V8 broth containing 50 μg/ml G418.*

*^d^Homozygous Avr1b-1^sgR^ or mCherry knock-in transformants verified by PCR and sequencing.*

*^e^Ratio of silenced mutants compared with total homozygous transformants. Abundance of transcripts from the Avr1b-1 locus was evaluated by RT-PCR as in Figure 7D and Supplementary Figure S7.*

## Discussion

The diverse family of RXLR effectors are key virulence factors of *Phytophthora* and other oomycete plant pathogens. The *Avr1b-1* RXLR gene of *P. sojae*, as well as its *P. infestans* homolog *PiAvr3a*, has served extensively as models for the study of RXLR effector functions, including impacts on host immunity and mechanisms of entry into host cells (Wang and Wang, 2018). Recently, Fang and Tyler (2016) developed a *Phytophthora* CRISPR/Cas9-mediated genome editing system using an oomycete-specific nuclear localization signal and oomycete-specific sgRNA transcription machinery. This *Phytophthora* genome editing tool allows detailed functional analysis of RXLR effector genes through knock-out, knock-in, and complementation experiments that avoid the uncertainties inherent in gene silencing and ectopic gene overexpression strategies. Here, we have conducted a series of knock-out and knock-in experiments with the *Avr1b-1* gene of *P. sojae.* These experiments have confirmed previous conclusions about the function of *Avr1b-1*, have revealed some new features regarding its expression, and expanded the utility of the CRISPR/Cas9 system in *P. sojae*.

Previous characterization of *Avr1b-1* had relied on transient expression in soybean leaves (Dou et al., 2008a, b), ectopic expression in *P. sojae* transformants (Dou et al., 2008a), gene silencing in *P. sojae* transformants (Song et al., 2013), assays of purified proteins (Shan et al., 2004; Kale et al., 2010), and phenotypes of *P. sojae* strains in which *Avr1b-1* was naturally silenced or absent (Shan et al., 2004; Cui et al., 2012). Here, we used CRISPR/Cas9-mediated gene knock-outs and complementation experiments to unambiguously test the role of *Avr1b-1* in producing an avirulence phenotype in the presence of the soybean *Rps1b* resistance gene. In these experiments, to avoid the potential off-targeting effects on a gene, *Avh1*, that is 97.6% identical to *Avr1b-1*, we selected P7063, which lacks *Avh1*, to perform CRISPR-mediated gene knock-out analysis. The results showed that the deletion of *Avr1b-1* enabled mutants to infect soybean plants containing *Rps1b*, confirming that *Avr1b-1* is essential for avirulence activity. The results also verified that the loss of *Avr1b-1* did not compromise the pathogenicity of *P. sojae* transgenic strains.

The “all-in-one” CRISPR/Cas9 system (Fang et al., 2017) has been successfully applied in several different *Phytophthora* species except for *P. infestans* (van den Hoogen and Govers, 2018; Chen et al., 2019; Wang W. et al., 2019; Ochola et al., 2020; Zhang et al., 2020). In most cases, the efficiency of homozygous replacement events was low (<10%). Our results showed that the frequency of homozygous replacement events in the *Avr1b-1* locus could reach 45%, but it varied among the sgRNAs. For this reason, when targeting a new gene, we typically try three different sgRNAs. For *mCherry*, we tested three different sgRNAs and all exhibited around 20% frequency of homozygous replacement events.

To conduct complementation following a knock-out, an alternative selection marker is needed for screening transformants. Besides G418, two other antibiotic markers, hygromycin B and streptomycin, have been used as selection markers in transgenic *P. infestans* (Ah-Fong and Judelson, 2011). In our hands, *P. sojae* isolates could maintain growth in V8 media containing up to 200 μg/ml hygromycin B. Therefore, it was not suitable for the screening of *P. sojae* transformants. Compared with G418, streptomycin is less effective as well. Although GFP has also been used as a selection marker for overexpression of spCas9 (Fang and Tyler, 2017), it is not suitable for the first round of screening due to the low transformation frequency. Oxathiapiprolin was described as an alternative selection marker in the genetic manipulation of *Phytophthora* pathogens (Wang W. et al., 2019), but the lack of commercial chemical products limits its application. Here, we found that G418 could be reused as a selection marker if the original KO transformant had lost its G418 resistance, even if the original gene was still present but silent. In *P. sojae*, G418 resistance is rapidly lost once G418 selection is removed, especially if the transformant is new. Therefore, reuse of G418 selection is feasible for routine complementation *via* a second round of transformation with the pYF515 vector. However, this strategy is only available if *npt II* was not used to replace the original target gene.

In order to conduct complementation, it was necessary to produce an *Avr1b-1* gene that was resistant to the three sgRNAs used to target the native *Avr1b-1* gene. This was needed in case the *sgRNA* genes were still being expressed in the original KO transformant and also to enable direct replacement of the native *Avr1b-1* gene with modified *Avr1b-1* genes. To produce *Avr1b*^*sgR*^, resistant to the three sgRNAs, we introduced multiple synonymous substitutions into the sgRNA target sites in *Avr1b-1*. The particle bombardment transient expression system was employed to verify that the expression of *Avr1b*^*sgR*^ would produce a resistance response in *Rps1b* soybeans. The suitability of *Avr1b*^*sgR*^ for this purpose was confirmed by the successful recovery of *Avr1b*^*sgR*^ gene replacements from both KO and wild-type recipient strains, producing transformants avirulent on *Rps1b* plants.

*Avr1b-1* is an infection-induced RXLR effector gene, and *mCherry* or *Avr1b-mCherry* genes introduced into the *Avr1b-1* locus showed similar transcript levels during infection as that of *Avr1b-1* in wild-type P7063 (except for those that had undergone silencing). Moreover, Avr1b-mCherry fusion proteins produced by the knock-in transformants specifically accumulated around haustoria during infection, whereas uniform distribution of mCherry proteins was observed in the *Avr1b-1* KO transformant Δ114-1-7 which carried the *Avr1b-1* promoter, but no signal peptide or effector sequences. Liu et al. (2014) reported that Avr1b-mRFP expressed from a constitutive promoter also specifically accumulated around haustoria during infection. These data suggest that the signal peptide and perhaps other targeting signals of Avr1b may be responsible for this localization pattern. In our experiments, we could not detect the effector-mCherry fusion inside haustorial host cells. Whisson et al. (2007) and Liu et al. (2014) reported similar results.

An unexpected feature of our results was the recovery of large numbers of knock-in transformants in which the incoming transgene had become silenced. We noticed that several different incoming genes, including *mCherry*, *Avr1b^*sgR*^-AVI*, and *Avr1b^*sgR*^-mCherry*, were subject to silencing in this way. Silencing occurred whether the transformation recipient was Δ114-1-7, carrying *mCherry* at the *Avr1b-1* locus, or was the wild-type strain P7063. All incoming transgenes were embedded in the same upstream and downstream 1-kb flanking ends from the *Avr1b-1* locus. Numerous studies have shown that sense as well as antisense constructs can readily induce silencing of both ectopic transgenes and endogenous genes in *Phytophthora* species (van West et al., 1999, 2008; Vu et al., 2019). However, our results are unusual in that only a single copy of the transgene is inserted into the genome, at the *Avr1b-1* locus. Our results are also unusual because other studies have reported normal expression of incoming transgenes after gene replacements at other *Phytophthora* loci (Ma et al., 2017; Miao et al., 2020; Ochola et al., 2020).

Our observations raise the interesting question of whether the *Avr1b-1* locus is unusually susceptible to silencing, due possibly to transcripts from the incoming plasmid DNA and/or epigenetic disturbances resulting from the replacement of genomic DNA with a segment of plasmid DNA. Among natural isolates of *P. sojae*, the frequency of *Avr1b-1* silencing is also high. In a survey of 34 US isolates, 10 contained silent *Avr1b-1* genes (Shan et al., 2004), while in a survey of 28 Chinese isolates, 5 contained silent *Avr1b-1* alleles (Cui et al., 2012). Elevated levels of histone H3 Lysine27 (H3K27) trimethylation were observed at the *Avr1b-1* locus in a naturally occurring *Avr1b*-silenced strain (P6497) but not in an *Avr1b*-expressing strain (Wang et al., 2020). Furthermore, mutations in a gene responsible for H3K27 methylation resulted in loss of silencing of *Avr1b-1* (Wang et al., 2020). Thus, epigenetic changes likely underlie changes in the silencing state of *Avr1b-1*.

In summary, our findings extend the utility of CRISPR/Cas9-mediated genome editing for exploring gene functions in oomycetes, including transcriptional variability, while at the same time emphasizing the importance of confirming that incoming gene replacements are expressed normally.

## Data Availability Statement

The original contributions presented in the study are included in the article/Supplementary Material, further inquiries can be directed to the corresponding author/s.

## Author Contributions

BG, XL, and BT conceived the research and wrote the manuscript with contributions from all authors. BG, GS, WG, and JM performed the experiments. BG, JM, and QW performed data analysis. All authors have read and agreed to the published version of the manuscript.

## Conflict of Interest

The authors declare that the research was conducted in the absence of any commercial or financial relationships that could be construed as a potential conflict of interest.
