# Supplementary figures and images for: Transcriptional Variability Associated With CRISPR-Mediated Gene Replacements at the Phytophthora sojae Avr1b-1 Locus
Source: Front Microbiol. 2021 Mar 18;12:645331. doi: 10.3389/fmicb.2021.645331 (PMC8012851; doi:10.3389/fmicb.2021.645331)

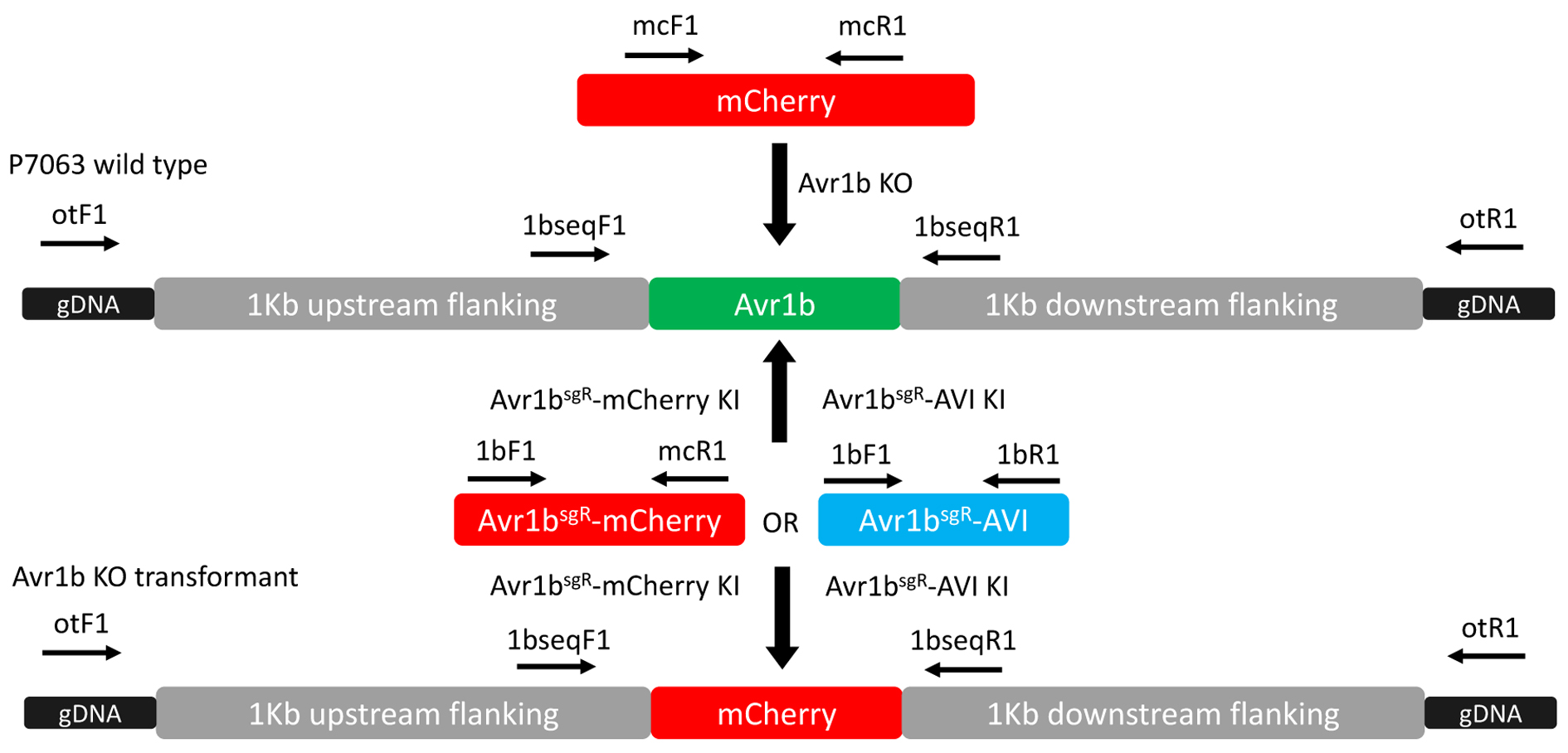

Supplement: Supplementary file 1 [file Image_1.JPEG]

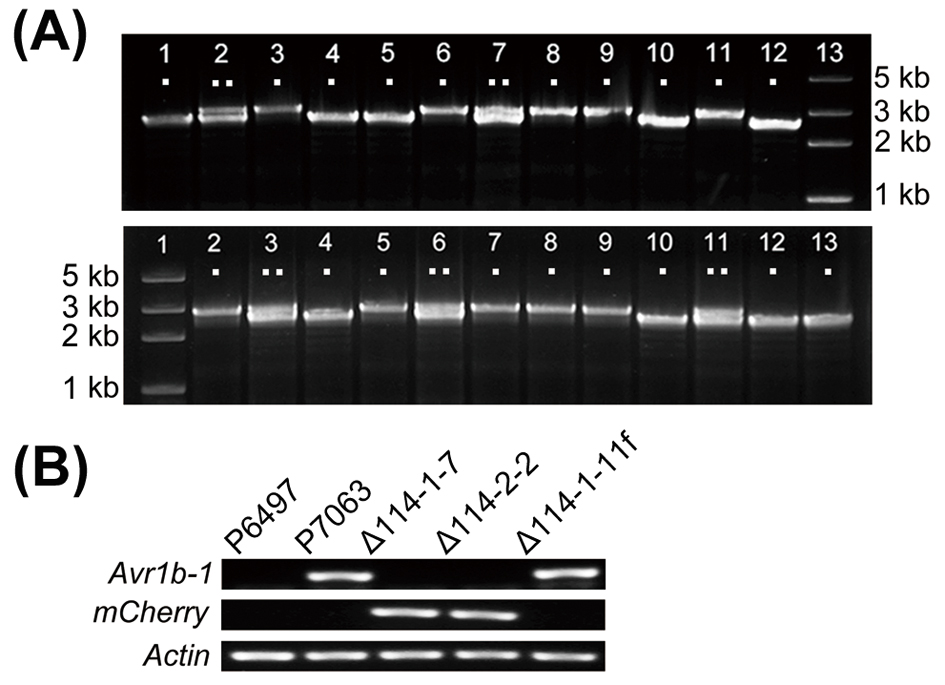

Supplement: Supplementary file 2 [file Image_2.JPEG]

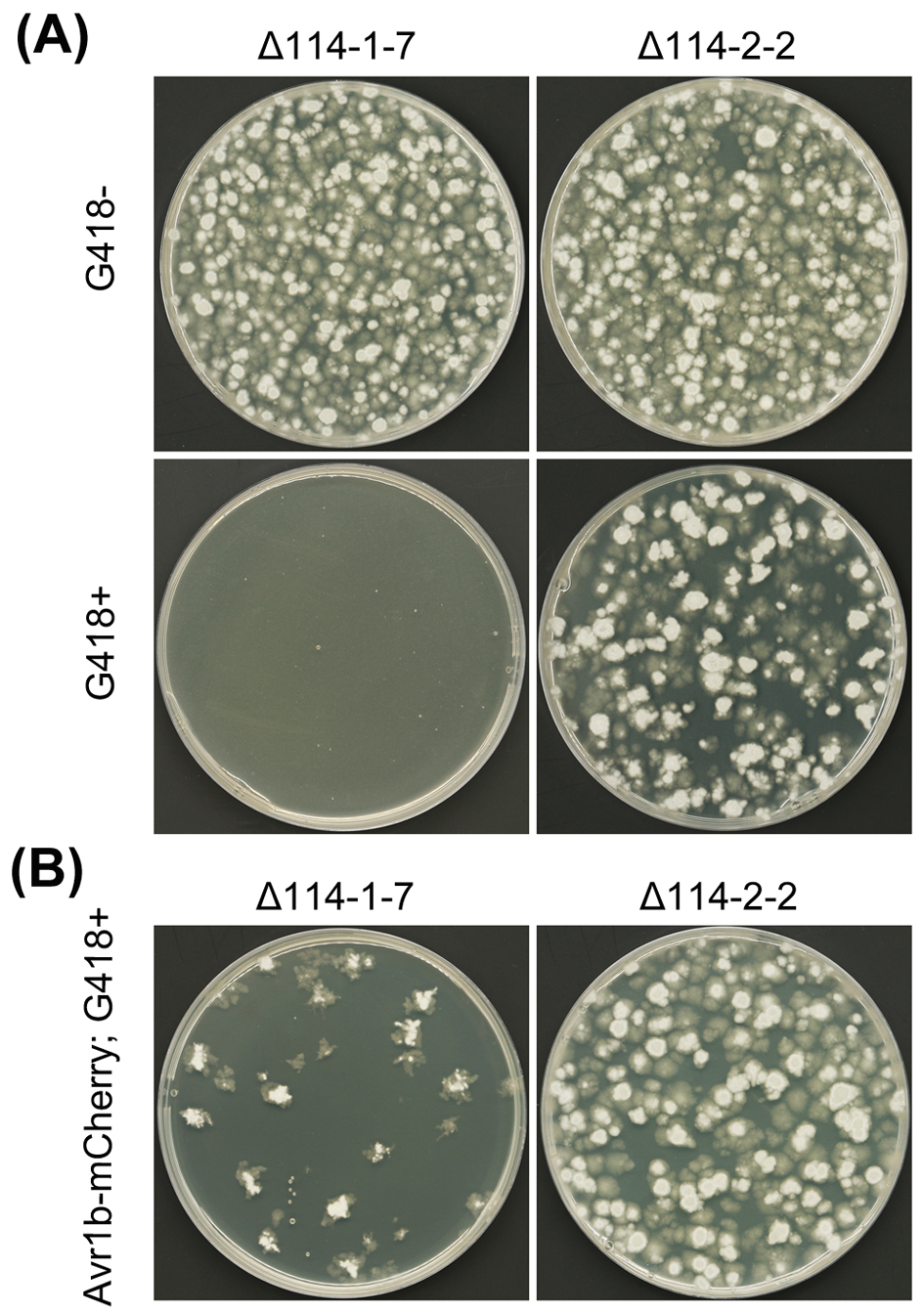

Supplement: Supplementary file 3 [file Image_3.JPEG]

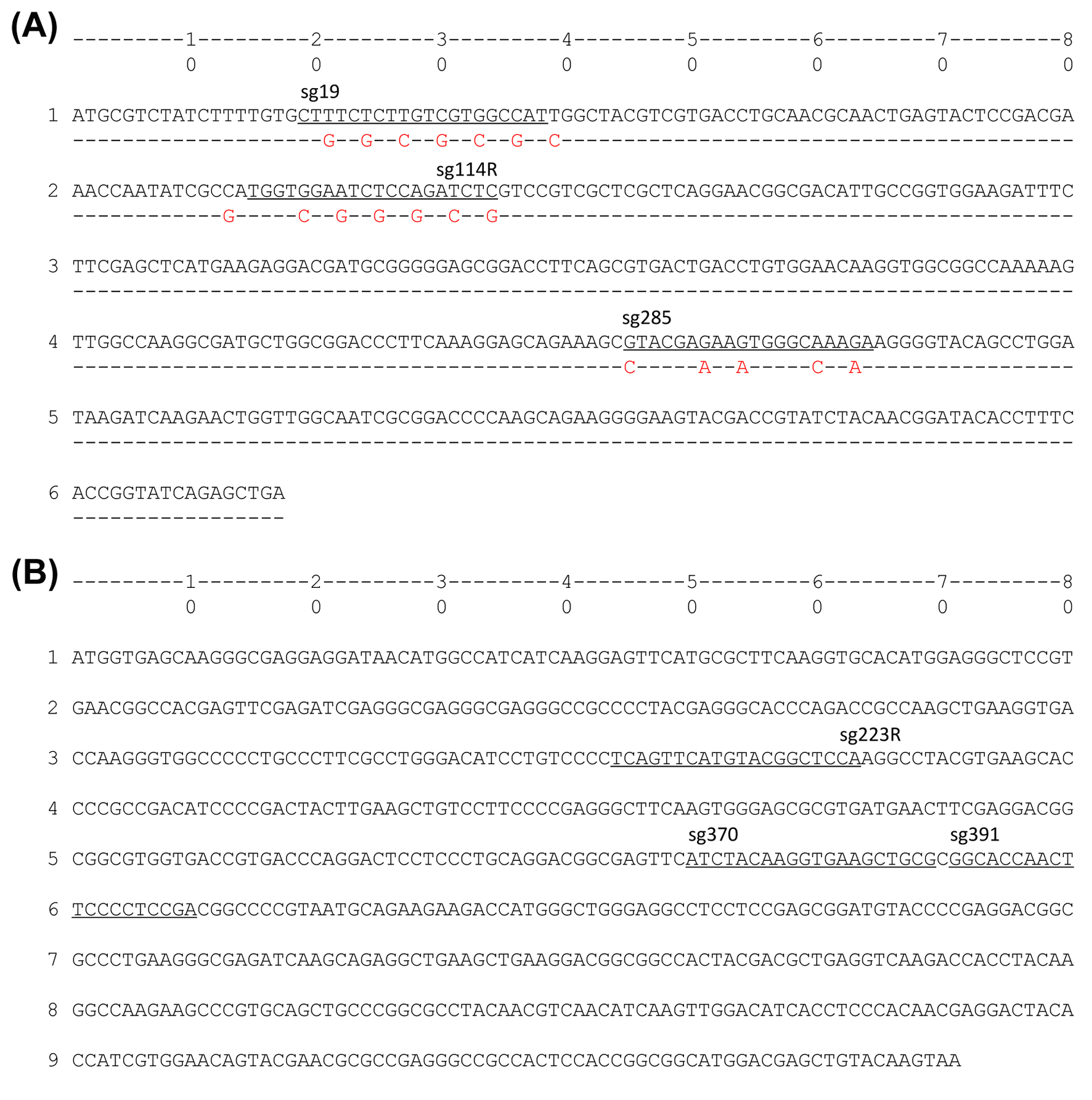

Supplement: Supplementary file 4 [file Image_4.JPEG]

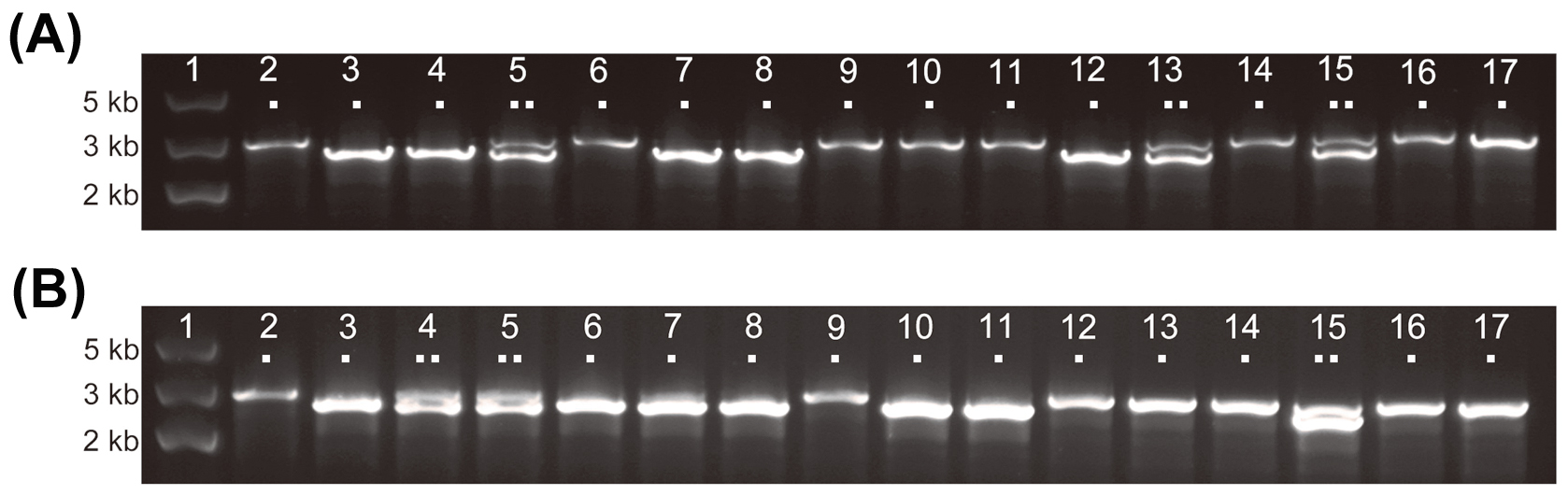

Supplement: Supplementary file 5 [file Image_5.JPEG]

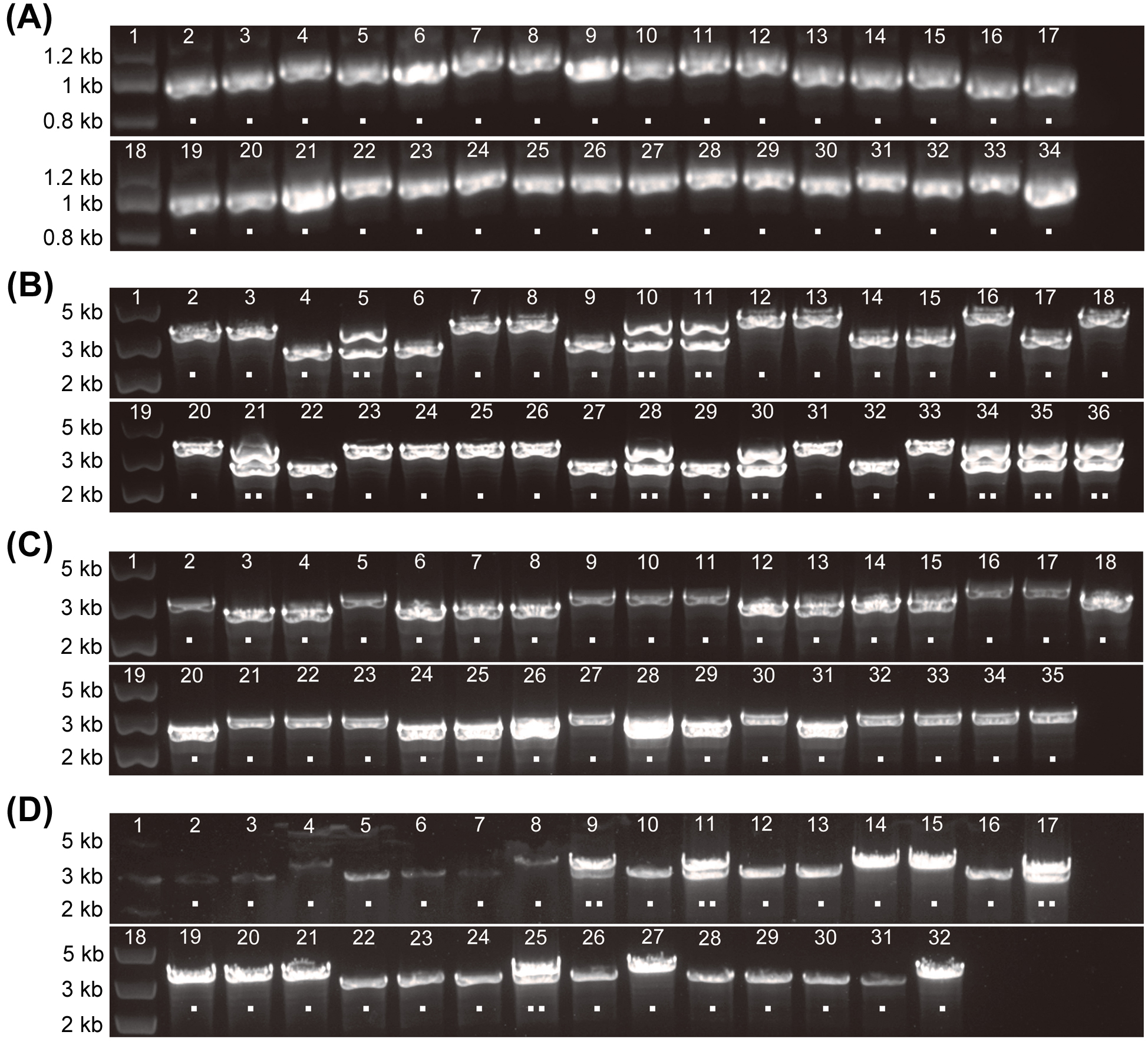

Supplement: Supplementary file 6 [file Image_6.JPEG]

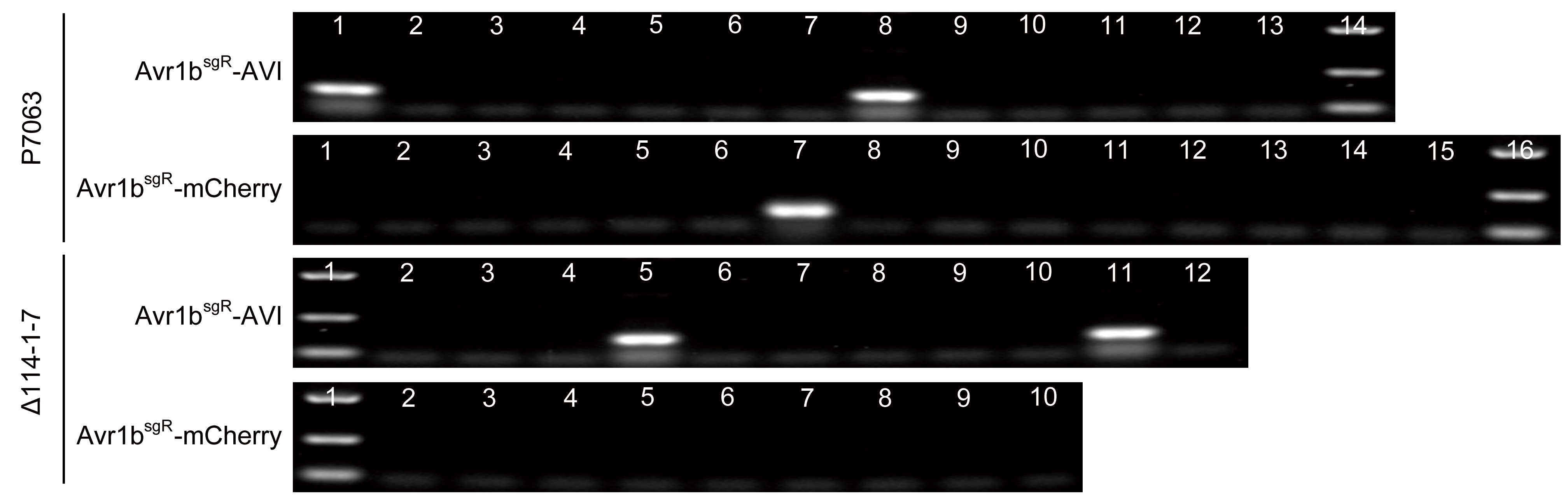

Supplement: Supplementary file 7 [file Image_7.JPEG]
