# Supplementary material for: Transcriptional Variability Associated With CRISPR-Mediated Gene Replacements at the Phytophthora sojae Avr1b-1 Locus
Source: Front Microbiol. 2021 Mar 18;12:645331. doi: 10.3389/fmicb.2021.645331 (PMC8012851; doi:10.3389/fmicb.2021.645331)
Supplement: Supplementary file 8 [file Data_Sheet_1.docx]

**SUPPLEMENTARY MATERIAL**

**Figure S1.** The strategy of CRISPR/Cas9-mediated gene replacement of *Avr1b-1*.Wild-type *P. sojae* P7063 genome was edited using CRISPR/Cas9 technology and the *Avr1b-1* coding region was replaced with *mCherry* or asgRNA resistant version of AVI- or mCherry tagged *Avr1b* to obtain HDR transformants. Either wild-type P7063 or the *Avr1b-1* knock-out (KO) transformant, Δ114-1-7, was used as the recipient for replacement with *Avr1b^sgR^*-AVI or *Avr1b^sgR^*-mCherry. The size of the left and right arms was 1 kb. Primer pairs otF1/otR1 and 1bF1/1bR1 were used to screen transformants.

**Figure S2.** PCR-based analysis of *Avr1b-1* knock-out mutants.**(A**) PCR screening of G418-resistant colonies produced by sg114R/Cas9-mediated mutagenesis using primerpair otF1/R1. Top panel (transformation 2): lane 1, wild-type P7063; lane2-11, *Avr1b-1* deletion transformants Δ114-2-1 to Δ114-2-11; lane 13, marker. Bottom panel (Transformation 1): lane 1, marker; lane 2-11, *Avr1b-1* deletion transformants Δ114-1-1 to Δ114-1-11; lane 13, wild-type P7063. White dots indicate if the *Avr1b-1* locus is homozygous (one dot) or heterozygous (two dots).**(B)**Semi-quantitative RT-PCR analysis of the *Avr1b-1* locus with primer q1bF/R or qmCheF1/R1. Δ114-1-7 and Δ114-2-2 are homozygous *Avr1b-1* KO transformants, Δ114-1-11f is a non-replacement transformant. Natural *Avr1b-1*-silenced isolate P6497 and *Avr1b*-expressing isolate P7063 were used as controls. *P. sojaeActin* was used as the reference gene.

**Figure S3**. Testing of *npt II*-silenced lines as potential transformation recipients. **(A)**Regenerated colonies of homozygous *Avr1b-1* knock-out transformants Δ114-1-7 or Δ114-2-2 growing in regeneration agar plateswith or withoutselection with 50 µg/ml G418. Pictures were taken after 3-days incubation.**(B)**Regenerated colonies of homozygous *Avr1b-1* knock-out transformants Δ114-1-7 or Δ114-2-2 growing in regeneration agar plates with 50 µg/ml G418for selection of *Avr1b-mCherry* overexpression transformants. Pictures were taken after 3-days incubation.

**Figure S4.** sgRNA binding sites of Avr1b and mCherry.Synonymous substitutions atsgRNA binding sites in the *Avr1b-1* coding sequenceare labeled in red.sgRNA binding sites in the Avr1b and the *mCherry* coding sequence were underlined. Sg114R of Avr1b and sg223R of mCherry binds to the antisenceDNA strand of target genes.

**Figure S5**. PCR-based screening of candidate *Avr1b^sgR^* knock-in transformants. **(A)** PCR screening of G418-resistant colonies derived from sg223R/Cas9-mediated knock-in with *Avr1b^sgR^* using primer pair otF1/R1. Top panel: lane 1, Marker; lanes 2-17, *Avr1b^sgR^* knock-in transformants C223-1 to C223-16. **(B)** PCR screening of G418-resistant colonies derived from sg391/Cas9-mediated knock-in with *Avr1b^sgR^*. Top panel: lane 1, Marker; lanes 2-17, *Avr1b^sgR^* knock-in transformants C391-1 to C391-16. White dots indicate if the *Avr1b-1* locus is homozygous (one dot) or heterozygous (two dots).

**Figure S6.** PCR-based screening of candidate *Avr1b^sgR^-AVI* and *Avr1b^sgR^-mCherry* knock-in transformants.**(A)**Nested PCR screening of G418-resistant colonies derived from sg114R/Cas9-mediated *Avr1b-AVI* knock-in and wild-type P7063using primer pairs otF1/R1 and 1bflF1/R1. Lanes 1 and 18, markers; lanes 2-17 and 19-34, *Avr1b-AVI* knock-in transformants. **(B)** PCR screening of G418-resistant colonies derived from sg114R/Cas9-mediated *Avr1b-mCherry* knock-in with -type P7063 using primer pair otF1/R1. Lanes 1 and 19, markers; lanes 2-18 and 20-36, *Avr1b-mCherry* knock-in transformants. **(C)** PCR screening of G418 resistant colonies derived from sg223/Cas9-mediated *Avr1b-AVI* knock-in with *Avr1b-1* knock-out mutant Δ114-1-7 using primer pair otF1/R1. Lane 1 and 19, markers; lane 2-18 and 20-35, *Avr1b-AVI* knock-in transformants. **(D)** PCR screening of G418-resistant colonies derived from sg223/Cas9 mediated *Avr1b-mCherry* knock-in with *Avr1b-1* knock-out mutant Δ114-1-7 using primer pair otF1/R1. Lanes 1 and 18, markers; lanes 2-17 and 19-32, *Avr1b-mCherry* knock-in transformants. White dots indicate if the *Avr1b-1* locus is homozygous (one dot) or heterozygous (two dots)

**Figure S7.** Levels of transcripts from the*Avr1b-1* locus in different replacement and complementation transformants during *in planta* growth.Homozygous *Avr1b^sgR^-AVI* or *Avr1b^sgR^-mCherry*transformants were used to inoculate Williams (*rps1b*) leaves. RNA samples were extracted from infected leaves at 48 hpiand used for RT-PCR analysis. Top panel, Lane 14, markers; lanes 1-13 *Avr1b^sgR^-AVI* replacement transformants with wild-type P7063 as the recipient. Second panel, lane 16, markers; lanes 1-15 *Avr1b^sgR^-mCherry* KI transformants. Third panel, Lane 1, markers; lanes 2-12 *Avr1b^sgR^-AVI* KI transformants with *Avr1b-1* deletion mutant Δ114-1-7 as the recipient. Bottom panel, Lane 1, markers; lanes 2-10 *Avr1b^sgR^-mCherry* KI transformants with *Avr1b-1* deletion mutant Δ114-1-7 as the recipient.

**Table S1.** The sequence of sgRNAs used for the gene replacement of *Avr1b-1* or *mCherry*

| Target | sgRNA^a^ | Sequence 5’-3’ |
| --- | --- | --- |
| *Avr1b-1* | sg19 | CTTTCTCTTGTCGTGGCCAT |
|  | sg114R | GAGATCTGGAGATTCCACCA |
|  | sg285 | GTACGAGAAGTGGGCAAAGA |
| *mCherry* | sg223R | TGGAGCCGTACATGAACTGA |
|  | sg370 | ATCTACAAGGTGAAGCTGCG |
|  | sg391 | GGCACCAACTTCCCCTCCGA |

a, sgRNAs sg19, sg285 and sg114R target *Avr1b-1* on the sense (+) and anti-sense (−) DNA strand; sgRNAs sg370, sg391 and sg223R target *mCherry* on the sense (+) and anti-sense (−) DNA strand.

**Table S2.** Characteristics of *PhytophthorasojaeAvr1b-1* deletion mutants at various developmental stage.

| Isolate^a^ | Colony Diameters (mm)^b^ | Sporangia  (No./field of view)^c^ | Zoospores  (No./field of view)^d^ | Germination of Cysts (%)^e^ | Oospores  (No./field of view)^f^ |
| --- | --- | --- | --- | --- | --- |
| P6497 | 26 ± 1 | 22 ± 3 | 42 ± 4 | 98 ± 1 | 198 ± 22 |
| P7063 | 23 ± 2 | 24 ± 5 | 36 ± 6 | 95 ± 2 | 189 ± 19 |
| Δ114-1-7 | 25 ± 2 | 22 ± 4 | 38 ± 5 | 97 ± 1 | 185 ± 14 |
| Δ114-2-2 | 23 ± 2 | 19 ± 5 | 32 ± 5 | 95 ± 2 | 201 ± 21 |
| Δ114-1-11f | 24 ± 2 | 25 ± 2 | 35 ± 3 | 98 ± 1 | 176 ± 31 |

a, Wild-type isolates P6497 and P7063; homozygous *Avr1b-1* knock-out transformants Δ114-1-7 and Δ114-2-2; failed KO transformant Δ114-1-11f. b, colony diameters were measured after 4 days’ culturing on 10% V8 plates. c, to analyze sporangia production, eight 4-mm plugs cut from the edge of fresh culture were placed into a petri dish with 20 mL of liquid 10% V8 medium. These plates were incubated 3 days in darkness at 25 ºC. After washing the mycelia several times using sterile water, another 15 mL of sterile water was added and incubated in darkness at 25 ºC for 24 h to induce sporangia production. The number of sporangia per field of view at 200x magnification formed on the plugs was counted under the microscope. d, to analyze zoospore production, all strains were cultured on 10% V8 liquid medium for 7-10 days in darkness at 25 ºC. Then, the plates were washed several times using sterile water. Finally, another 5 mL of sterile water was added and the plates were incubated at 25 ºC for 4-8 h. e, cyst germination was assessed by plating about 300 zoospores on 10% V8 agar medium. After 4-6 h of incubation in the dark at 25 ºC, the percentage germination of each strain was observed by counting 100 cysts. Each experiment contained three replica plates and all experiments were repeated 2 times. f, For oospore production, the mycelia was cultured on 10% V8 agar for 7 days. Then, oospores were counted using the light microscope at 200x magnification. The number was the means of random ten fields of view. The mean ± standard deviation. No statistical differences were observed for any of the characteristics by the Dunnett’s test.

**Table S3.** Primers used in this study.

| **Primers** | **Sequence 5’-3’** |
| --- | --- |
| Fl-Avr1b | GATAAGCTTGATATCgaattCTGTGGCTACATGTGAGAG |
| Rl-Avr1b | GATAGACGCATGGTTCGTGATACTTGGCGAC |
| Fr-Avr1b | TAAGAAGGTCTCGCCGAAATCG |
| Rr-Avr1b | CGCTCTAGAACTAGTggatcCACCCAACGAAAAAAACACTG |
| otF1 | CTCGAGCCATCACTCAAGGT |
| otR1 | GTTTAGAAGAGCGGCTCTTG |
| 1bF1 | GAGTCTAGCGCAGCCATACC |
| 1bR1 | TGCCAATACCACCAAGTTGA |
| 1b-F | CTAGCCCCGGGATGCGTCTATCTTTTGTGC |
| 1b-R | CTGAGGTACCTCAGCTCTGATACAGGTGAA |
| 1bseqF | ATCCCTTGTCGTTCTGTTGC |
| 1bseqR | AGGCTGGGAGTGACGTATTG |
| q1bF | GCGTGACTGACCTGTGGA |
| q1bR | TGAAAGGTGTATCCGTTGTAG |
| qPsActinF | TCTGGCACCACACGTTCTAC |
| qPsActinR | CGTAACCCTCGTAGATGGGC |
| qNptF | TCTTTTTGTCAAGACCGACCTGT |
| qNptR | GCCACAGTCGATGAATCCAGAAA |
| qmCheF1 | CTCCTCCGAGCGGATGTACC |
| qmCheR1 | GGTGGTCTTGACCTCAGCGT |
| sgR19F | ctagcAGAAAGCTGATGAGTCCGTGAGGACGAAACGAGTAAGCTCGTCCTTTCTCTTGTCGTGGCCAT |
| sgR19R | aaacATGGCCACGACAAGAGAAAGGACGAGCTTACTCGTTTCGTCCTCACGGACTCATCAGCTTTCTg |
| sgR114F | ctagcGATCTCCTGATGAGTCCGTGAGGACGAAACGAGTAAGCTCGTCGAGATCTGGAGATTCCACCA |
| sgR114R | aaacTGGTGGAATCTCCAGATCTCGACGAGCTTACTCGTTTCGTCCTCACGGACTCATCAGGAGATCg |
| sgR285F | ctagcTCGTACCTGATGAGTCCGTGAGGACGAAACGAGTAAGCTCGTCGTACGAGAAGTGGGCAAAGA |
| sgR285R | aaacTCTTTGCCCACTTCTCGTACGACGAGCTTACTCGTTTCGTCCTCACGGACTCATCAGGTACGAg |
| PsF | GCCAAGTATCACGAACCATGCGTCTATCTTTTGTGCTG |
| PsR | GATTTCGGCGAGACCTTCTTAGCTCTGATACCGGTGAAAGG |
| Ps4WTR1 | CTCGATCTTCTGGGCCTCGAAGATGTCGTTCAGGCCGCTCTGATACCGGTGAAAG |
| Ps4WTR2 | CGATTTCGGCGAGACCTTCTTACTCGTGCCACTCGATCTTCTGGGCCTCG |
| 1bmcR | TCCTCCTCGCCCTTGCTCACGCTCTGATACCGGTGAAAG |
| 1bmcF | CCTTTCACCGGTATCAGAGCGTGAGCAAGGGCGAGGAGGAT |
| R-mcR | GCATGGACGAGCTGTACAAGTAAGAAGGTCTCGCCGAAATCG |
